# Unveiling Uncommon Manifestations in a Pediatric Patient With Systemic Lupus Erythematosus: A Case Report

**DOI:** 10.7759/cureus.20418

**Published:** 2021-12-14

**Authors:** Raksha Ranjan, Sonalika Mehta, Kanchan n Saxena

**Affiliations:** 1 Pediatrics-Pediatric Nephrology, All India Institute of Medical Sciences, Rishikesh, IND; 2 Pediatrics, All India Institute of Medical Sciences, Rishikesh, IND

**Keywords:** endocarditis, mononeuritis multiplex, vasculitis, antiphospholipid syndrome, systemic lupus erythematosus

## Abstract

Systemic lupus erythematosus (SLE) is a chronic autoimmune disease with multi-organ involvement. It may involve skin, kidneys, joints, central nervous system (CNS), and cardiopulmonary system. Marked variations in clinical presentations are seen in SLE patients, ranging from subclinical to life-threatening manifestations. SLE and antiphospholipid syndrome (APS) may be associated with Libman-Sacks endocarditis. Visceral vasculitis usually manifests with disease ﬂares and can affect almost any organ. APS can have arterial or venous thrombosis and the presence of persistently positive antiphospholipid antibodies (aPL), including lupus anticoagulants (LA), anticardiolipin antibodies (aCL), and/or anti-β2-glycoprotein-I antibodies (aβ2GPI). Peripheral neuropathy is unusual in pediatric patients.

We present a case of an adolescent girl with juvenile SLE (JSLE) in whom endocarditis and digital gangrene at first presentation were actually masquerading underlying life-threatening secondary APS with extensive medium vessel thrombosis. Additionally, there was cutaneous and visceral vasculitis and a rare peripheral nervous system (PNS) manifestation, mononeuritis multiplex (MNM).

## Introduction

Systemic lupus erythematosus (SLE) is a highly complex disease with an unpredictable natural history. The Systemic Lupus International Collaborating Clinics (SLICC) requires patients to either meet ≥4 of 17 criteria, including at least one clinical and one immunological criterion, or have biopsy-proven lupus nephritis with positive antinuclear antibody (ANA) or anti-double-stranded DNA (anti-dsDNA) to establish a diagnosis of SLE [1].

## Case presentation

Our patient was a 17-year-old school-going adolescent girl, a native of a northern state in India. She presented in a very sick state with complaints of fever for 20 days, an ulcer on the right thumb and ring finger and left foot fourth toe for 15 days, and difficulty in breathing for seven days. She had undergone tests outside (echocardiography showing endocarditis, blood culture revealing methicillin-sensitive Staph aureus) one week prior. At presentation, she had a weak and rapid pulse, tachypnea, blood pressure near the fifth centile, and SpO_2_ of 99%. She also had a high-grade fever. On head-to-toe examination, there was pallor, skin necrosis, and gangrene of digits of the right hand and left foot. On systemic examination, there were bilateral (B/L) diminished breath sounds, gallop rhythm, with a pansystolic murmur, moderate hepatomegaly, and moderate splenomegaly. The left fundus examination revealed an old hemorrhage. Hematological and radiological investigations are presented in Table 1.

**Table 1:** Hematological and radiological workup of the patient TLC: total leucocyte count; CECT: contrast-enhanced computed tomography

Tests	Results
Hemoglobin (HB)	6 gm/dl
TLC	31,800 cells/cmm
Polymorphonuclear cells	86%
Lymphocytes	9%
Platelet count	2.56 lakh/cmm
Peripheral blood film	No evidence of hemolysis
CRPh	213 mg/dl
Chest radiograph	B/L pleural effusion (PE)
CECT thorax	Empyema
CECT abdomen	Splenic abscess
Echocardiography	9-mm vegetation on anterior mitral valve

The patient's kidney and liver function tests and urine analysis were within normal limits, and tropical infection-related workup was negative. She had no joint pain, malar rash, photosensitivity, or chest pain. Central nervous system (CNS) examination at the time of presentation was unremarkable. She had a history of thrombocytopenia one year prior, attributed to immune thrombocytopenic purpura (ITP). There was no history of similar illnesses in the family. After stabilization, her left radial pulse was found to be feeble compared to the right. Echocardiography showed 9-mm vegetation over the anterior mitral valve, and arterial Doppler revealed the left axillary, left common iliac, and proximal external iliac artery thrombosis.

We considered the possibility of antiphospholipid syndrome (APS)/connective tissue disorder like SLE, and the evaluation was initiated accordingly. Prothrombin time/international normalized ratio (PT/INR), activated partial thromboplastin time (APTT), anti-thrombin III (63.3%), protein C (66.1%), protein S (82.7%), fibrinogen (386.7 mg/dl), and lipid profile were all in the normal range; direct and indirect anticoagulation tests were negative; ferritin was elevated at 2,000 ng/ml, and rapid plasma reagin (RPR) was non-reactive. Table 2 shows the immunological profile of the patient.

**Table 2:** Immunological profile ANA: antinuclear antibody; dsDNA: double-stranded DNA; IgG: immunoglobulin G

Tests	Results
ANA	Positive with nuclear-speckled pattern 2+
Lupus anticoagulant	Positive
dsDNA	Negative
Anticardiolipin IgG	Negative
Anticardiolipin IgM	Negative
C3	87 (low)
C4	18.8 (normal)

Her condition improved symptomatically with culture-sensitive antibiotics, drainage of infective foci, anti-CCF measures, anticoagulants (initially dabigatran later warfarin), and packed red cell transfusion. She was started on hydroxychloroquine and steroids. At the one-month follow-up, her left radial pulse flow and skin lesions were found to be improved, and vegetations over the mitral valve had disappeared. However, anemia (normocytic normochromic) persisted, with normal platelet count and normalized total leukocyte count. Furthermore, she had now developed subacute-onset pain in the left leg and foot, which was associated with numbness. On examination, there was tenderness over the dorsum of the left foot and loss-of-touch sensation over the left big toe. There was a weakness in the dorsiflexion of the left foot and foot drop. Simultaneous nervous system examination did not reveal neurological deficits at other nerve distribution sites. The nerve conduction study showed peripheral neuropathy localizing to the left peroneal nerve, B/L median and ulnar nerve, and B/L sural nerve suggestive of mononeuritis multiplex (MNM) (Table 3).

**Table 3:** Nerve conduction study

Upper limbs
B/L distal motor latencies of the median nerve comparable to the ulnar nerve
B/L F wave latency of the median and ulnar nerve - within normal limits
B/L sensory latency of median and ulnar - not recordable
Lower limbs
Motor and F wave latencies of left peroneal - could not be recorded
Increased motor latencies of the left tibial nerve
Normal F wave latency of B/L tibial nerve
Sensory latency of B/L sural nerve could not be recorded

## Discussion

Our patient had positive lupus anticoagulants (LA), ANA, hypocomplementaemia, serositis, and MNM, thereby fulfilling the SLICC criteria for SLE. We present this case to highlight the treacherous presentation of not-so-common clinical features of juvenile SLE (JSLE).

There are no validated criteria for the diagnosis of pediatric APS [2]. In the pediatric APS Registry, APS in children was associated with SLE in 39%. In a retrospective study involving 58 Chinese children with APS, ANA positivity and hypocomplementaemia were more commonly found in APS associated with SLE. LA, anticardiolipin antibodies (aCL), and anti-β2-glycoprotein-I antibodies (aβ2GPI) were positive in 95%, 64%, and 77% of the cases, respectively. The index case had LA positivity along with ANA positivity and hypocomplementaemia. With single-positive LA, the proportion with venous thrombosis was 100% [3]. Potentially life-threatening catastrophic APS occurs in <1% cases and has a high mortality rate characterized by tissue thrombotic events occurring in a short period with histopathological evidence of small vessel thrombosis and laboratory confirmation of the presence of antiphospholipid antibodies (aPL) [4]. Cardiac valve disease [5] or intra-cardiac thrombosis [6] can be the initial manifestations of pediatric APS. Primary APS is associated with a younger age at onset and a higher frequency of arterial thrombotic events. In contrast, APS associated with underlying autoimmune disease is linked to older age at onset.

Vasculitis is an integral part of disease activity assessment tools for SLE. But the incidence and prevalence of vasculitis in JSLE have not been well described. Vasculitis in JSLE commonly presents as cutaneous vasculitis rather than visceral vasculitis, affecting the heart, large vessels, lungs, CNS, peripheral nervous system (PNS), gut, and kidneys. A study involving 179 patients with JSLE in the UK found that 12% experienced cutaneous vasculitis [7]. Symptoms of vasculitis can be non-specific, and presentations depend on the size of vessels involved. Cutaneous vasculitis most frequently affects the limbs [8]. Cutaneous ulcers, nodules, digital gangrene, livedo racemosa, and pyoderma-gangrenosum like lesions indicate arterial involvement. Individuals affected have a higher probability of associated visceral vasculitis [9]. In SLE, pulmonary presentations of vasculitis are acute lupus pneumonitis and diffuse alveolar hemorrhage [10]. Cardiac vasculitis in JSLE typically comprises pericarditis, cardiomegaly, valvulitis, and conduction abnormalities. The most common causes of extremity gangrene in SLE are APS and endarteritis [11]. Differentiating vasculopathy/thrombosis and vasculitis in SLE-associated APS can be difficult and requires histological assessment of cutaneous lesions.

SLE is the most common rheumatic disease associated with MNM [12]. MNM may be an initial manifestation of visceral vasculitis in adult SLE [13], but it is extremely uncommon in pediatric SLE. Only two cases were seen among 3119 electromyography studies performed in patients less than 19 years of age at the Children’s Hospital, Boston, between 1979 and 2003. A countable number of cases have been reported in the literature and only one case has been reported from India in the last 10 years in the pediatric age group [14]. MNM affects two or more non-contiguous nerves simultaneously or sequentially [15], and results from a vasculitic insult to vasa nervosum leading to Wallerian degeneration of nerve fibers [16]. A positive association of aPL with the possibility of development of this type of vasculitic neuropathy is also suggested. Mononeuropathy usually is a late complication; the diagnosis of SLE preceded the onset of MNM by a mean (SD) of 75 (98) months in a cohort study [12]. Contrary to our case where MNM was seen almost simultaneously at diagnosis, it is in fact one of the clinical criteria included to establish a diagnosis. In the index case, the possibility of MNM due to APS in the underlying SLE cannot be ignored.

APS coexisting with SLE is commonly associated with vasculitis, a manifestation of which can be MNM. The term mononeuritis multiplex, by definition, requires multiple nerves to be affected; but the involvement and expression of symptoms of neuropathy in different topographies can be sequential. Our patient had just started to complain of leg pains and was subjected to a nerve conduction study suggestive of MNM due to the development of weakness. The patient has not developed symptoms of neurological deficits outside the left peroneal nerve distribution to date. 

Vasculitic neuropathy/MNM, when associated with a systemic autoimmune disease, is typically linked to active systemic disease; however, in the index case, dsDNA and ENA profile was negative. Moreover, despite the clinical interest in and numerous publications on biomarkers in SLE, there is no validated and widely accepted biomarker for flare prediction in SLE to date. In a systematic review by Gensous et al. [17], none of the newly studied biomarkers stood out, and the routinely performed biomarkers appeared to be deceiving, with contradictory results.

Vasculitic manifestations like MNM and digital gangrene with APS in our patient signify a more active and aggressive disease. Our case emphasizes the need to review and reassess our understanding of the not-so-common clinical manifestations of JSLE, which are the only manifestations at times.

## Conclusions

Endocarditis, along with thrombosis, can be a presenting feature of JSLE. A high index of suspicion for severe secondary APS is required in such cases. One should be vigilant for PNS manifestations, and routine nerve conduction studies may have a role in detecting subclinical cases.
